# Dose Optimization in TOF-PET/MR Compared to TOF-PET/CT

**DOI:** 10.1371/journal.pone.0128842

**Published:** 2015-07-06

**Authors:** Marcelo A. Queiroz, Gaspar Delso, Scott Wollenweber, Timothy Deller, Konstantinos Zeimpekis, Martin Huellner, Felipe de Galiza Barbosa, Gustav von Schulthess, Patrick Veit-Haibach

**Affiliations:** 1 Department of Medical Imaging, Nuclear Medicine, University Hospital Zurich, Zurich, Switzerland; 2 GE Healthcare, Waukesha, Wisconsin, United States of America; 3 Department of Medical Imaging, Diagnostic and Interventional Radiology, University Hospital Zurich, Zurich, Switzerland; Banner Alzheimer's Institute, UNITED STATES

## Abstract

**Purpose:**

To evaluate the possible activity reduction in FDG-imaging in a Time-of-Flight (TOF) PET/MR, based on cross-evaluation of patient-based NECR (noise equivalent count rate) measurements in PET/CT, cross referencing with phantom-based NECR curves as well as initial evaluation of TOF-PET/MR with reduced activity.

**Materials and Methods:**

A total of 75 consecutive patients were evaluated in this study. PET/CT imaging was performed on a PET/CT (time-of-flight (TOF) Discovery D 690 PET/CT). Initial PET/MR imaging was performed on a newly available simultaneous TOF-PET/MR (Signa PET/MR). An optimal NECR for diagnostic purposes was defined in clinical patients (NECR_P_) in PET/CT. Subsequent optimal activity concentration at the acquisition time ([A]_0_) and target NECR (NECR_T_) were obtained. These data were used to predict the theoretical FDG activity requirement of the new TOF-PET/MR system. Twenty-five initial patients were acquired with (retrospectively reconstructed) different imaging times equivalent for different activities on the simultaneous PET/MR for the evaluation of clinically realistic FDG-activities.

**Results:**

The obtained values for NECR_P_, [A]_0_ and NECR_T_ were 114.6 (± 14.2) kcps (Kilocounts per second), 4.0 (± 0.7) kBq/mL and 45 kcps, respectively. Evaluating the NECR_T_ together with the phantom curve of the TOF-PET/MR device, the theoretical optimal activity concentration was found to be approximately 1.3 kBq/mL, which represents 35% of the activity concentration required by the TOF-PET/CT. Initial evaluation on patients in the simultaneous TOF-PET/MR shows clinically realistic activities of 1.8 kBq/mL, which represent 44% of the required activity.

**Conclusion:**

The new TOF-PET/MR device requires significantly less activity to generate PET-images with good-to-excellent image quality, due to improvements in detector geometry and detector technologies. The theoretically achievable dose reduction accounts for up to 65% but cannot be fully translated into clinical routine based on the coils within the FOV and MR-sequences applied at the same time. The clinically realistic reduction in activity is slightly more than 50%. Further studies in a larger number of patients are needed to confirm our findings.

## Introduction

Positron emission tomography/magnetic resonance (PET/MR) using 18-Fluoro-deoxyglucose (FDG) opens potentially new perspectives in the field of clinical molecular imaging. Combining the high soft tissue contrast of MR, functional image procedures of MR and the molecular ability of PET may improve the anatomical correlation and provide more clinically relevant information [1]. As a new imaging modality, it is hoped that PET/MR shows some significant advantages over PET/Computed tomography (CT) e.g. in head and neck cancer evaluation [2,3] or liver metastases detection [4]. Besides the potential of improved lesion characterisation, one expected benefit is the reduction of radiation exposure by omitting the CT–based dose and also reducing the FDG- activity requirement[5]. Such a reduction in activity in the PET-component of the evaluated TOF-PET/MR system can be achieved e.g. with a combination of silicon-based detector technology and larger solid angle coverage [6,7].

The noise-equivalent count rate (NECR) represents an objective measurement of PET-system performance that reflects the ratio of true events to the overall detected events, which include randoms and scatters [8]. It is calculated following the National Electrical Manufacturers Association (NEMA) recommendations, using a 20-cm-diameter and 70-cm-long cylinder that is assumed to provide a reasonable characterization of whole-body image quality [9].

The noise equivalent count rate is the standard metric for PET scanner performance provided by the manufacturers and determined as part of acceptance testing for new equipment [10].

However, obtaining the NECR values from phantoms does not entirely reflect clinical routine behavior in patients since this measurement simply does not account for variations in the fractions of the scatter and random events that are internal to the patient [11].

In a pre-evaluation to this presented study, it was shown that NECR measured in patients can predict clinically perceived image quality in PET-imaging and a corresponding FDG-activity threshold above which the acquired PET images have good-to-excellent perceived quality in more than 90% of patients [12].

Using these data as the basis of the present study, we use the NECR measured in patients in PET/CT to predict the theoretically achievable FDG-activities in a new whole-body TOF-PET/MR, based on the NECR curves measured in a standard NEMA phantom.

The aim of the study was (1) to investigate the amount of *theoretically* possible dose reduction in a new TOF-PET/MR compared to standard TOF-PET/CT by comparison of NECR-measurements in patients (in PET/CT) and NECR measured in phantoms (PET/MR) and (2) to evaluate the *clinically realistic* reduction in activity in PET/MR by evaluation of different (retrospectively reconstructed) imaging times which are equivalent to different FDG-activities.

## Materials and Methods

### Patient population

A total of 75 consecutive patients (81 exams) were evaluated retrospectively. All patients were referred for a clinical FDG-PET/CT from January to December/2012 and underwent a PET/CT-MR using a tri-modality setup. Based on the retrospective nature of the study, no formal institutional ethics committee approval was needed and a waiver was provided.

Exclusion criteria applied were uncontrolled glucose levels and patients who did not fast for a minimum of 4 hours prior to the examination, unwillingness to undergo the additional MR examination, claustrophobia, MR-incompatible medical devices (e.g. cardiac pacemaker, insulin pump, neurostimulator, cochlear implant). Another exclusion criterion was presence of artefacts in at least one bed-position, which made clinical reading not applicable. Parts of the evaluated patient population were also evaluated within the context of another study which is currently under review as well.

### PET/CT imaging

PET/CT imaging was performed on a PET/CT-MR setup including a time-of-flight Discovery 690 PET/CT and a Discovery 750w 3T MR (both GE Healthcare, Waukesha, WI).

PET/CT was performed according to the EANM procedure guidelines for tumour PET imaging [13]. Patients fasted for at least 4 hours prior to injection of ^18^F-fluorodeoxyglucose (FDG). The mean FDG injected activity was 311.7 MBq (SD = 21.3 MBq, range 231.1–373.4 MBq). The mean FDG injected activity/body weight was 4.3 MBq/kg (SD = 0.9 MBq/kg, range 2.6–6.6 MBq/kg).

Unenhanced low-dose CT and PET emission data were acquired from the mid-thigh to the vertex of the skull. CT data were acquired in shallow-breathing with dose modulation between 15–80 mA, 120 kVp and a pitch of 0.984:1, reconstructed to images of 0.98 mm transverse pixel size and 3.75 mm slice thickness. PET data was acquired in 3D time-of-flight mode with scan duration of 2 min per bed position, an axial FOV of 153 mm and a 23% overlap of bed positions, resulting in a total PET acquisition time ranging from 16 to 20 minutes. The emission data were corrected for randoms, dead time, scatter and attenuation and iteratively reconstructed (OSEM, 3 iterations, 18 subsets)[3].

### Image processing and analysis

The acquired PET and CT images were transmitted to a dedicated review workstation (Advantage Workstation, GE Healthcare), which enables the review of the PET and CT images side by side or in fused/overlay mode (PET/CT).

In a first step, all data were automatically analysed using Matlab (MathWorks, Natick, MA, USA) to estimate the noise-equivalent count rate (NECR) versus activity concentration in a similar manner to the National Electrical Manufacturers Association (NEMA) analysis, including scatter or attenuation correction methods applied to patient data and how scatter and randoms were estimated, based on previous publications [11,14–17]. This measurement is essentially the same usually performed on phantom data, but the required data (the number of true, scattered and random counts) are estimations either provided by the scanner software or automatically extracted from the reconstructed image. Methods how to evaluate NECR measurements in patients have been described before by different authors [8,11,15]. To highlight the fact that these NECR values were measured on patient data, we indicate this as NECR_P_.

In a second step, all the PET/CT exams were read by a board-certified nuclear medicine physician / radiologist and by a radiologist with substantial experience in PET/CT image reading.

One subjective score was defined to evaluate image quality. The *IQ local score* (IQ_L_) was a three-point scale assigned to each bed position in axial plane in all patients (n = 655 bed positions), where 1 means poor, 2 means good and 3 means excellent IQ. In general, smoothness and sharpness of the images have been considered for the evaluation. The liver homogeneity and graininess and the contrast between different structures that accumulate different levels of tracer, like the lung and chest wall, were analysed.

Patient data were analysed concerning overall weight, body mass index (BMI) and FDG-activity at the start of acquisition (D_Acq_). One additional parameter was defined for each patient: the ratio between D_Acq_ and patient weight (R_DW_).

**Fig 1:**
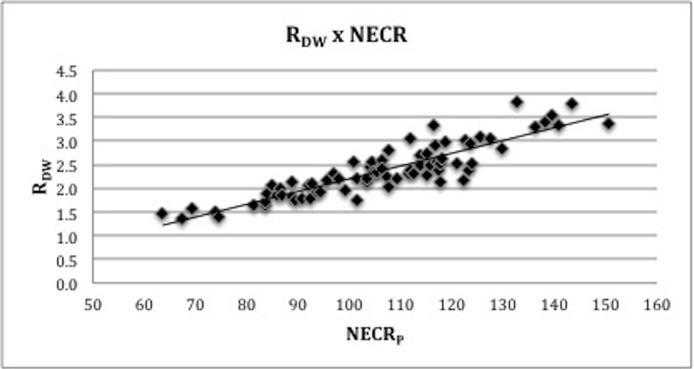
Relation between R_DW_ and R_BMI_ and clinical NECR measured in patients (NECR_P_).

The R_DW_ threshold was determined, above which the resulting image quality was at least “good” (IQ_L_ > 2) in more than 90% of patients. The rationale for choosing R_DW_ as the threshold parameter is that this measurement is routinely used to calculate patient’s dose, according to EANM guidelines, making it more reproducible. Using the R_DW_ threshold, the optimal NECR_P_ for diagnostic purposes was defined based on the R_DW_ x NECR_P_ curve (Fig 1).

**Fig 2:**
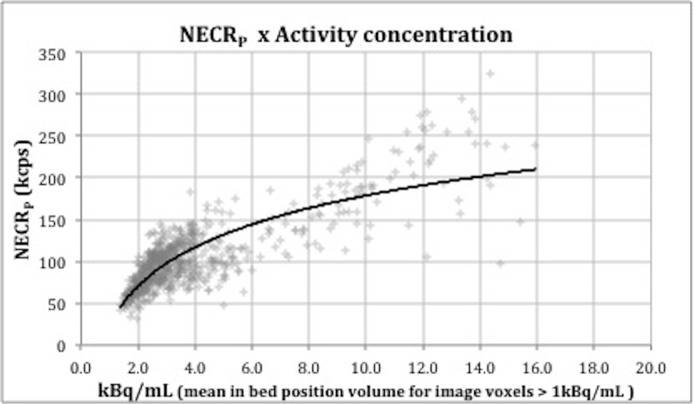
Relation between NECR measured in patients and activity concentration.

**Fig 3:**
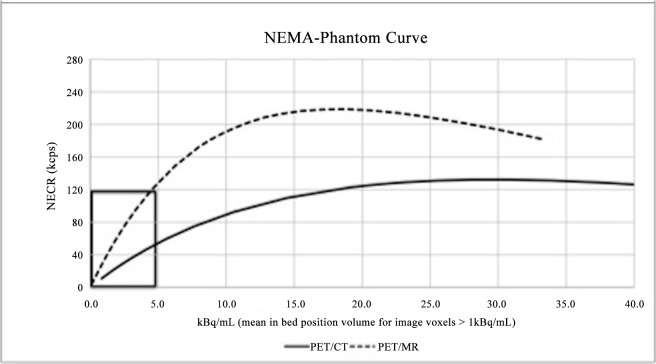
The NEMA phantom curve of the TOF-PET/CT (continuous) and TOF-PET/MR (dashed), provided by its manufacturer. See enlarged image of the clinically relevant area (black box) in Fig 4.

The NECR_P_ was then cross-referenced in the graphic NECR_P_ x Activity concentration (Fig 2) in order to get the corresponding optimal activity concentration at the acquisition time ([A]_0_). By obtaining this value, it is now possible to establish the relation between patient-based and phantom-based count-rate measurements. Applying the calculated [A]_0_ value on the NEMA phantom curve of the GE Discovery D690 (Fig 3) that was used in these acquisitions, the target NECR (NECR_T_) was obtained. Notice that this image quality target value is independent of the PET system used.

Using this NECR_T_, it is therefore possible to estimate, for any PET system, the activity concentration required to obtain the desired image quality (e. g. good-to-excellent”) in a given percentage of patients ([A]_N_), by cross-evaluation with the NECR curves of the system compared.

In a pre-evaluation a threshold in which 90% of cases were rated with “good to excellent” image quality was defined [12].

In the presented study, this procedure was performed for the particular case of a new whole-body TOF-PET/MR device in order to predict the injected FDG- activity needed to provide good-to-excellent images, which is expected to be significant lower. The GE Signa PET/MR is a whole-body scanner combining a 3T wide-bore MR system with a 25 cm PET detector ring based on SiPM technology and mounted on a customized radiofrequency coil.

Furthermore to transfer our results not only technically but also clinically, the first 25 patients on the new simultaneous TOF-PET/MR were evaluated as well. For this purpose, (acquired 2 min/bed in PET/CT and 4 min/bed in simultaneous TOF-PET/MR) patient-data was reconstructed retrospectively to expose both systems to the same amount of *emitted* counts. Reconstructed acquisition times were adjusted for tracer decay between the acquisitions.

The acquisition times were reconstructed to be similar by unlisting the list mode data and creating similar sinograms to be compared for each acquisition time. This way, the two resulting sinograms are representing an equivalent of the same injected ^18^F-FDG acitivity. After reducing PET/MR acquisition times to match the PET/CT decay integral, the average was 196 ± 25 seconds/bed.

Our next step was to reduce both, PET/CT and PET/MR acquisition times in pre-defined steps to evaluate at which time point the PET/CT and/or the PET/MR image were considered not “good to excellent” anymore. For these whole body scans, we un-listed the emission data for 120, 100, 80, 60, 40 and 30 seconds/bed for PET/CT; the corresponding equivalent times for PET/MR were 206, 170, 134, 98, 68, 53 ± 25 seconds/bed. By defining this threshold, and again applying the calculated value on the NEMA phantom curve of the simultaneous PET/MR system, it is possible to extrapolate not only a theoretical “technical” threshold for activity reduction but also a clinically validated threshold.

### Statistical Analysis

All statistical tests were performed using SPSS Statistics Version 21 (IBM, Armonk, NY, USA). *P*-values < 0.05 were considered statistically significant. Pearson’s correlation analysis was performed to evaluate the relation between NECR, and R_DW_.

## Results

The R_DW_ was significantly correlated with NECR_P_ (r = 0.89, p-value < 0.01), as presented on Fig 1. The threshold at scan time calculated for R_DW_ was 2.6 MBq/kg.

The obtained values for NECR_P_, [A]_0_ and NECR_T_ were 114.6 (± 14.2) kcps, 4.0 (± 0.7) kBq/mL and 45 kcps, respectively. Cross-referencing this NECR_T_ to the phantom curve of the new TOF-PET/MR system (Fig 3), the corresponding activity concentration at scan time was found to be approximately 1.4 kBq/mL. At this activity concentration, 90% of images would be rated with “good to excellent” image quality.

Thus, the theoretical activity concentration required by the new TOF- PET/MR is 35% of the activity concentration required by the TOF- PET/CT. The corresponding ideal activity threshold at the injection time is 1.3 MBq/kg.

With the same method it is possible to calculate and find any required clinical image quality threshold as well as the required threshold for the injected activity for any system for which the NECR curve is available. Considering 3.8 MBq/kg as the optimal threshold at injection time for the compared TOF-PET/CT, which requires an activity concentration of 4.0 kBq/mL, the optimal threshold could be calculated with T_O_ = 0.95 x [A]_N_, where the [A]_N_ is the activity concentration value for which the corresponding NECR is 45 kcps in the NECR curve.

**Fig 4:**
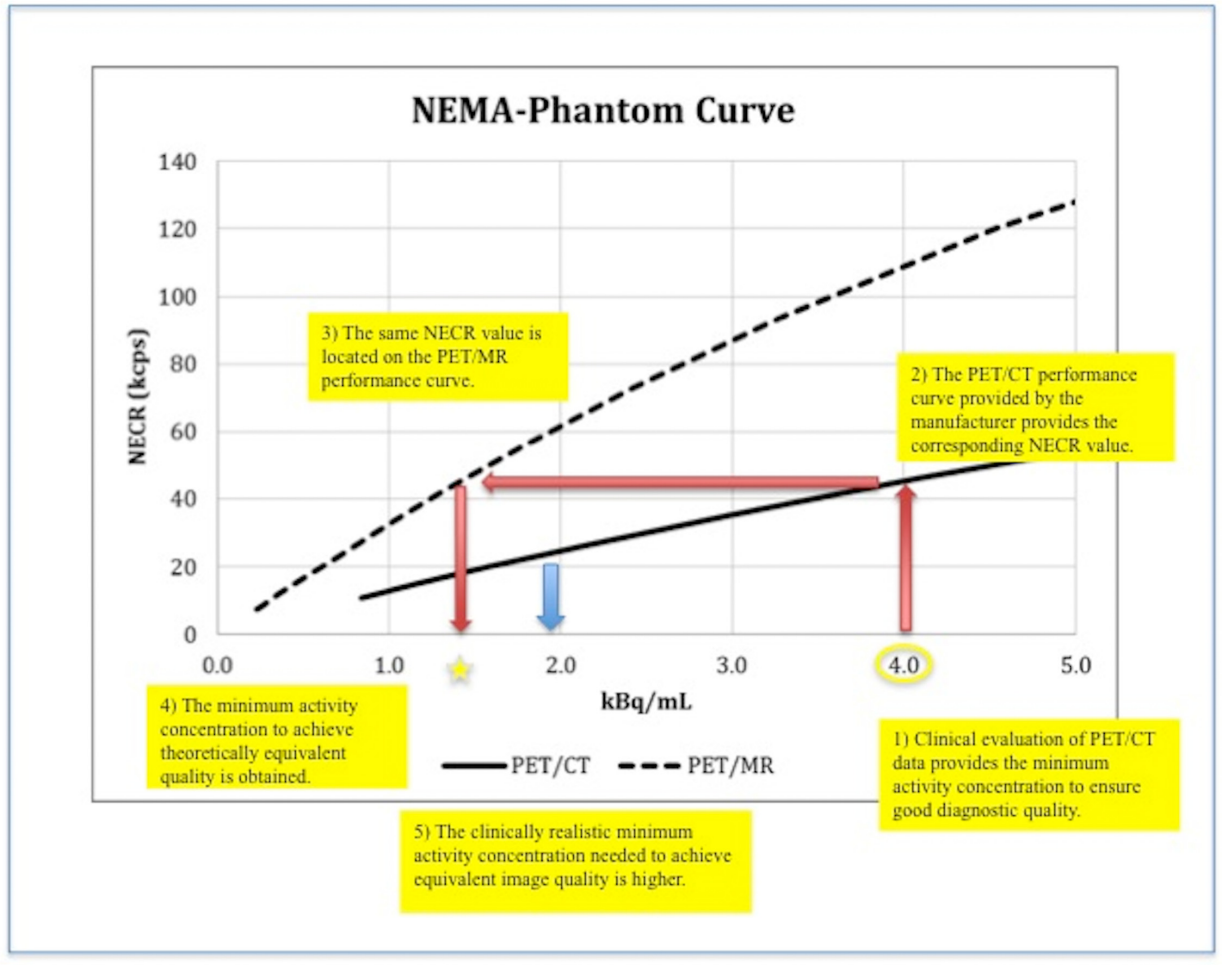
Evaluation and transfer steps from activities in PET/CT (1) to theoretically (2,3) achievable reduction in injected activity in TOF-PET/MR (4) and clinically realistically activities in TOF-PET/MR (5—blue arrow).

Since these values are representing the theoretically possible reduction in injected activity, the evaluation on the simultaneously acquired PET/MRI showed slightly different results. Here, on average the PET/MR image was rated not “good to excellent” anymore at a time point equivalent to 1.8 kBq/mL which represents 44% of the initially required activity (Fig 4).

## Discussion

This study is an extension of a previous study about clinical image quality perception and corresponding NECR measurements in PET systems. The results of this pre-evaluation indicated that findings based on the NECR_P_ metric could be rescaled and applied for different PET systems [8,11,15,18]. Other groups have evaluated methods about NECR measurements in patients and corresponding image quality as well [8,19,20]. In the presented study, to the best of our knowledge, we are the first to present the extrapolation of clinically evaluated image quality and corresponding NECR measurements in patients from PET/CT to a new TOF-PET/MR (SIGNA PET/MR, GE Healthcare, Waukesha, MI, USA). Our results show that with this new system, a theoretical reduction in injected activity down to nearly one third of ^18^F-FDG, and a clinically realistic reduction of slightly more than 50% in FDG-activity compared to a TOF-PET/CT is possible.

Much effort has been put into optimization of the injected FDG-activity and maintaining image quality at the same time. Ideally, FDG-activity should be kept as low as possible considering the cost of the tracer and the radiation exposure for the patient and staff [21]. Patient morphology, the time acquisition per bed position and the used PET reconstruction (e.g. 2D vs. 3D, time of flight) are some of the parameters that have been shown to influence the FDG-dosing requirements [11,16,21].

However, regardless all of these parameters, the performance of every PET scanner is intrinsically (and non-linearly) dependent on the amount of injected activity. Although there is a debate on how to derive NECR values, the measurements following the NEMA protocol represent one of the most accepted and widely used way of showing the relation between the activity and acquired data quality. The controversial issue concerning the use of NECR is the difficulty to extrapolate the phantom-based measurements to patient acquisitions, since the response in humans can be quite variable [11]. The use of NECR nevertheless appears as a potential tool to standardize the FDG activity needed to get images in diagnostic quality. One possibility is to measure the NECR directly in clinical patients, which has already been done in other studies [8,11].

Recently, McDermott et. al have shown that the noise equivalent counts per axial length was also an effective and objective indicator of patient image quality, being able to discriminate images of good/excellent quality from those of poorer image quality with higher degree of accuracy than noise equivalent count density and liver signal to noise ratio [22].

Another challenge of correlating the NECR measured in patients to the visually perceived PET-image quality assessed by imaging specialists. Previous studies have compared the NECR phantom-based to visual image quality [17,19] or NECR measured in patients to signal-to-noise as a parameter of image quality [8,11].

As an initial step, our study compared the NECR measured in patients and visual image quality assessed by nuclear medicine physicians/radiologists, favouring the clinical reliability of our findings compared to other available studies that were not assessed by imaging specialists.

In a second step, the newly available TOF-PET/MR has been evaluated using the NEMA recommendations. A NECR curve (phantom based) has been created and was used to estimate the theoretically required activity concentration in order to achieve again good-to-excellent images. In the last step these results were then evaluated and transferred to the simultaneously acquired PET/MR images to evaluate the clinically realistic reduction in injected activity.

Our findings suggest that the optimal activity concentration required in the PET/MR is 35% (a reduction of 65%) of the activity concentration required by the comparatively used TOF-PET/CT. There are several technical reasons and explanations for this.

Delso et. al have already suggested that the integrated PET/MR features could require lower activities[5]. For a non-TOF whole-Body PET/MR used by Delso et al., a longer axial FOV and reduced detector ring diameter lead to higher count rates and an increased sensitivity, both in stand-alone operation and with simultaneous MR image acquisition. Thus, such a PET/MR system reaches its saturation and dead-time points with lower activities[5].

The evaluated TOF-PET/MR system has an increased axial field of view (FOV) compared to the TOF-PET/CT, too. Thus, the increased axial FOV increases sensitivity at the scanner center in a d^2^ (axial field of view) relationship. For example, going from 15 to 25cm axial FOV leads to (25/15)^2^, which is a 2.78 factor improvement.

Another feature of the evaluated system scanner is the diameter compared to standard PET/CT, which also provides an approximately linear gain in sensitivity. For example here, going from 810 mm to 622 mm diameter leads to (810/622) a factor of 1.3 of improvement. The evaluated PET/MR has a detector face-to-face diameter of 62 cm and an axial FOV of 25 cm in PET.

Concerning detector configuration, the new PET system employs five rings of 112 detector blocks where the scintillator crystals are coupled to 1 x 3 arrays of SiPM devices. The SiPM itself has several advantages compared with conventional detector materials, e.g. timing resolution, gain and corresponding noise [23,24].

The PET detectors modules are mounted on the outside of a novel radiofrequency (RF) body coil that provides additional space to accommodate the PET detector ring with a 60 cm patient bore. This design also reduces the amount of PET-attenuating material in the PET field of view, contributing in part to the reduced activity requirements. The use of scatter recovery techniques additionally supports this advantage as well [25].

A recent study has evaluated the advantage of the implementation of such recovery procedures by using the inter-block Compton scatter on TOF-PET scanners. It was found that this setup is expected to result in an overall scanner sensitivity improvement of up to 20% without significant processing overhead [25].

Summing up all these newly embedded technologies in the evaluated TOF-PET/MR, the analysed system has the potential to require significantly less activity while preserving diagnostic image quality.

However, there are also several reasons to consider why those improvements in dosage are not fully translated into clinical routine. To make full use of the system’s capabilities, the full field-of-view has to be used to achieve the full sensitivity. This is obviously not possible for brain imaging where the “natural” field-of-view is shorter.

Furthermore, one has to account for the MR-coils within the PET-FOV, which causes a decrease in counts as well. This is especially important for brain imaging, because the head/neck coils are a fully surrounding cage, while surface coils for body imaging are made of less material and are more flexible [26–29]. Thus, in cases of brain imaging, one might loose up to 15–20% of sensitivity in the center of the image based on the head and neck coil. Surface coils however, account for a smaller amount of sensitivity loss [30].

Lastly, the execution of MR-sequences are known to decrease the performance of PET-systems, too, however, largely not clinically significant (< 5%) [31]. Summing up these additional considerations, an activity reduction of up to 65% (or using just 35% of the activity compared to current TOF-PET/CT) is a theoretical number, but clinical reality as initially evaluated here also shows roughly a reduction in injected activity of > 50%.

One limitation is certainly that we only evaluated the first initial patients on the simultaneous PET/MR to test the clinical transferability of our results.

Furthermore, clinical image perception is always prone to a certain extent of subjectivity of the readers; clinical image perception in PET/MRI might be different compared to PET/CT.

## Conclusion

The new TOF-PET/MR device requires significantly less activity to generate PET-images with good-to-excellent image quality, due to improvements in detector geometry and detector technologies. The theoretical achievable reduction of ^18^F-FDG acitivity was proven based on cross-evaluation of clinical images quality in PET/CT and phantom based NECR measurements in TOF-PET/MRI. Further studies in a larger number of patients are needed to confirm our findings.
